# Insistence on sameness, repetitive negative thinking and mental health in autistic and non-autistic adults

**DOI:** 10.1177/13623613241275468

**Published:** 2024-09-14

**Authors:** Kate Cooper, Ailsa Russell

**Affiliations:** Centre for Applied Autism Research, University of Bath, UK

**Keywords:** anxiety, autism spectrum disorders, depression, interventions – psychosocial/behavioural, psychiatric comorbidity, quality of life, repetitive behaviours and interests

## Abstract

Autistic people experience high rates of mental health problems. A tendency towards behavioural repetition in autistic individuals could contribute to repetitive negative thinking, which is a transdiagnostic risk factor for mental health problems. Our research aimed to add to the literature by using transdiagnostic measures of both mental health symptoms and repetitive negative thinking. We explored the relationships between insistence on sameness and depression-anxiety, via the mediating role of repetitive negative thinking, operationalised as rumination and obsessing. Three groups of adults were recruited; autistic clinical cohort participants (*n* = 67); autistic community participants (*n* = 54) and non-autistic community participants (*n* = 66). All participants completed measures of Insistence on sameness; Anxiety and Depression; Obsessing; Rumination. Autistic community participants had significantly higher insistence on sameness, rumination and obsessing scores than non-autistic community participants. The mediation analysis found support for the hypothesis that the relationship between insistence on sameness and depression-anxiety would be mediated by rumination and obsessing in all participants. The higher rates of repetitive negative thinking found in autistic individuals may contribute to elevated rates of mental health problems in this group. Mental health assessments and interventions for autistic people should consider transdiagnostic factors including multiple forms of repetitive negative cognition and behaviour.

**Lay abstract:**

Autistic people are more likely to have mental health problems than non-autistic people. We know that having repetitive and negative thoughts can contribute to multiple mental health problems such as depression and obsessive-compulsive disorder. Autistic people often do the same behaviours repetitively, and they may also have more repetitive thinking styles. This could contribute to higher rates of mental health problems in autistic people. In this research, we wanted to find out if higher rates of repetitive behaviours contributed to depression and anxiety, and whether this relationship was because of repetitive negative thinking. We asked three groups of autistic adults to take part in the research. Sixty-seven *autistic clinical* participants were recruited from clinical settings and had moderate depression. Fifty-four *autistic community* participants and 66 *non-autistic community* participants were recruited from community settings. All participants completed measures of repetitive behaviours; a measure of anxiety and depression; and two measures of repetitive negative thinking (ruminating and obsessing). Autistic community participants had significantly higher repetitive behaviours, rumination and obsessing scores than non-autistic community participants. We found that higher rates of repetitive behaviours contributed to more repetitive thinking (obsessing and ruminating), which contributed to higher rates of depression and anxiety. The higher rates of repetitive negative thinking found in autistic individuals may contribute to higher rates of mental health problems in this group. Mental health assessments and interventions for autistic people should therefore consider the role of multiple forms of repetitive negative cognition and behaviour, which cut across diagnostic categories such as anxiety, depression and obsessive-compulsive disorder. These should be considered when aiming to understand why individuals develop mental health conditions and why these conditions persist.

Autism, a neurodevelopmental condition characterised by difference in social communication and a restricted, repetitive pattern of behaviours, interests and activities is present in approximately 1% of people (Zeidan et al., 2022). Autistic people are at significant risk of experiencing mental health problems, with a lifetime prevalence of 42% for anxiety conditions and 37% for depression (Hollocks et al., 2019) and high rates of obsessive-compulsive disorder (OCD; e.g. Lai et al., 2019). Multiple genetic and psychosocial factors contribute to the elevated rates of anxiety and depression in this group (Han et al., 2022; Nord & Garfinkel, 2022; Rydzewska et al., 2019). In terms of psychological factors, high rates of repetitive cognitive processes such as worry and rumination have been found to be associated with anxiety and depression in autistic people (DeLong, 2004; Gotham et al., 2014; van Heijst et al., 2020). Repetitive cognitive processes have typically been investigated in respect to specific mental health problems and are under-investigated as a transdiagnostic process in mental health problems in autistic people.

Repetitive negative thinking (RNT), prevalent across several emotional disorders, is considered a transdiagnostic factor. RNT describes a thinking process that is repetitive, negative in content and difficult to disengage from (Ehring & Watkins, 2008). RNT is associated with multiple mental health conditions including depression, generalised anxiety disorder (GAD) and OCD. As a transdiagnostic factor, there are similarities in the process of RNT across different conditions, but some disorder-specific differences in the content or form of RNT (e.g. Wahl et al., 2019). For example, rumination or RNT about the self which is past-orientated (Trapnell & Campbell, 1999) has links with depression in typically developing populations (Smith & Alloy, 2009). Worry, described as ‘a chain of thoughts and images, negatively affect-laden and relatively uncontrollable’ (Borkovec et al., 1983, p. 9) is a prominent feature of GAD. While the hallmark of OCD is persistent and recurring intrusive thoughts, images or impulses which cause distress (Diagnostic and Statistical Manual of Mental Disorders, Fifth Edition (*DSM*-5), American Psychiatric Association, 2013), ruminative thinking is common in OCD and distinct from obsessional rumination (Wahl et al., 2011). In OCD, obsessional rumination refers to intrusive unwanted (obsessional) thoughts without observable compulsive behaviour, although neutralising internal rituals may be present. Rumination about OCD, that is, attempting to cope with obsessions by analysing the causes and consequences of obsessional thoughts is conceptually distinct. Such rumination may in turn exacerbate OCD symptoms by strengthening negative internal appraisals of obsessional thoughts (Freeston & Ladouceur, 1997; Raines et al., 2017).

A pattern of repetitive, restricted behaviours, interests or activities is an important domain of autism (5th ed.; *DSM*–5; American Psychiatric Association, 2013), and this includes a wide range of phenomena from stereotyped motor movements to focused interests. A subcategory of the restricted repetitive behaviours (RRBI) domain of autism is *insistence on sameness* (Bishop et al., 2013), which refers to a preference for routine, difficulties with change and compulsive or ritualistic behaviour. A tendency towards repetitive routines could lead to lower mood and higher anxiety. For example, preferred routines and resistance to minor changes may manifest as an autistic individual engaging in an activity in an unvarying fashion with resistance to and distress about potential change, that is, needing to watch recordings of past television programmes in a specific order each day when returning home. This routine may not only leave little time and space for new activities, but also potentially reduce the positive attributes of the preferred activity over time, with an impact on mood. Similarly, a preference for sameness may reduce opportunities to challenge and overcome anxiety in new, unfamiliar activities and environments, for example, it is less likely that an individual will engage in a different activity at that point in the daily routine, consider alternative material to view or easily tolerate delaying the routine for an alternative activity, for example, if on holiday. Following periods of a narrow behavioural repertoire, unfamiliar or new activities may become associated with anxious and fearful response patterns, fostering avoidance of change.

While these proposed accounts are consistent with behavioural models of depression and anxiety, they are speculative. Studies have found relationships between insistence on sameness, repetitive sensory motor behaviours and anxiety in autistic children (Wigham et al., 2015), suggesting that insistence on sameness plays a significant role in anxiety for autistic populations. In a study of parent ratings of 72 autistic children and young people, high rates of ritualistic and sameness behaviours were significantly associated with severity of anxiety, depressive and oppositional defiant disorder symptoms (Stratis & Lecavalier, 2013). This latter study controlled for adaptive function, which was shown to moderate the relationship between stereotypical motor behaviours and psychiatric symptoms. There has been less research investigating the role insistence on sameness may play in co-occurring mental health problems for autistic adults, and particularly for depression.

While the behavioural models outlined have credence, cognitive theories and the mediating role of cognitions are considered central to modern conceptualisations of emotional disorders (Beck, 2002). It is therefore plausible that the relationships between the behavioural features of autism and emotions such as mood and anxiety may need to be extended to include cognitive factors associated with autism such as reduced cognitive flexibility (Demetriou et al., 2018), focused thinking styles (Happé & Frith, 2006) and repetition at the cognitive level (Cooper et al., 2022). Measures of RRBI are predominantly informant measures of observable behaviours developed for use with parents of autistic children or clinician ratings. A more recently developed measure (the adult Repetitive Behaviour Questionnaire or RBQ-2A; Barrett et al., 2015) based on earlier informant versions of the repetitive behaviour questionnaire captures adults’ self-report of RRBI including insistence on sameness. This measure has been designed for use in both autistic and non-autistic adults, with a similar factor structure across groups, indicating that this can be a useful transdiagnostic measure (Brett et al., 2024). However, the RBQ-2A does not enquire about cognitive repetition. In an earlier study, we used descriptive experience sampling methods (DESM; Cooper et al., 2022) to investigate cognitive repetition. We found that a community sample of autistic adults reported significantly greater numbers of repeated thoughts when compared with non-autistic people. The autistic people in the study did not report significantly more negative thoughts. Self-reported insistence on sameness on the RBQ-2A was not associated with numbers of repeated thoughts in either group. However, in the autism group, higher ratings on the insistence on sameness measure were associated with higher scores on a measure of obsessional thinking and total OCD symptoms. These findings led us to conclude that while repetition in autism may extend to perseverative cognition, in and of itself, cognitive repetition may not account for the increased levels of emotional disorders reported in autism. Other facets of repetitive cognitive processes, which were not captured by the DESM may be critical in understanding the associations between autism repetition and emotional disorders.

RNT may then be an important transdiagnostic factor in investigations of the elevated levels of depression, anxiety and OCD for autistic as well as non-autistic individuals. Autistic adults indeed report engaging in more rumination than non-autistic adults, and rumination has been found to significantly correlate with depression scores in autistic people (Crane et al., 2013). In autistic adolescents, rumination has been found to correlate with depression scores, as well as insistence on sameness (Gotham et al., 2014). In a study of these phenomena in a sample of 200 non-autistic people but capturing the broader autism phenotype using the AQ, Keenan et al. (2018) found that perseverative thinking was associated with rumination and depression. However, scores on the autism quotient were not a moderator of the relationship between perseverative thinking and rumination. Network analysis of rumination and depression measures involving a large sample of autistic adults found strong associations between the different facets of rumination and the depression symptoms of sadness and guilt were important bridge nodes in the overlap between rumination and depression (Williams et al., 2021). A recent longitudinal study identified that trait-like RNT at baseline was significantly associated with both depression and anxiety symptoms (measured separately) over the course of 12 weeks in autistic and non-autistic college students (McKenney et al., 2023). In addition, one study in 762 autistic adults found that insistence on sameness had a moderate relationship with depression and that this was attenuated by the inclusion of neuroticism in the regression analysis, indicating that personality factors are important in the relationship between repetitive behaviour and depression (Schwartzman et al., 2022).

In summary, RNT, including obsessional thinking, requires further investigation as a transdiagnostic factor for emotional disorders in neurodiverse groups. There is some evidence from studies of autistic children and non-autistic adults that repetition at the behavioural and cognitive levels may be associated with RNT and with anxiety. There is some evidence that RNT may be a significant factor in depression and anxiety for autistic adults. We sought to investigate the role of insistence on sameness (i.e. behavioural repetition) and RNT in depression and anxiety for both autistic and non-autistic adults. The aims of our study were to recruit a neurodiverse sample including autistic and non-autistic adults (1) to investigate if RNT is a transdiagnostic factor in emotional disorders and (2) to investigate if RNT mediates the association between insistence on sameness and emotional disorders.

We hypothesised that

1. A preference for routine (insistence on sameness) will be higher in autistic compared to non-autistic individuals.
2. RNT operationalised as rumination and obsessing scores will be higher in autistic compared to non-autistic individuals.
3. In the full neurodiverse sample (autistic and non-autistic groups), higher levels of insistence on sameness will predict higher depression-anxiety scores, and RNT (i.e. rumination and obsessing) will mediate these relationships.

## Method

### Participants

Adults aged 18 years and older (*n* = 187) were recruited to participate in this study (see Table 1 for demographic information). Participants were recruited in three groups, during the course of two separate studies. All autistic participants had a diagnosis of an autism spectrum disorder, with diagnostic reports checked by the study team to ensure they reported a valid clinical diagnosis of autism from a health professional.

**Table 1.** Demographic variables by group.

Characteristic	Autistic				Non-autistic	
	Clinical cohort		Community		Community	
	*N*	%	*n*	%	*n*	%
Gender						
Female	16	24	20	37	38	58
Male	49	73	32	59	27	41
Other	2	3	2	4	1	2
Ethnicity						
White	65	97	50	94	62	94
Asian	2	3	1	2	2	3
Mixed race	0	0	2	4	2	3
Relationship status						
Single	43	64	30	56	26	39
Cohabiting/married	16	24	14	26	19	29
In a relationship	7	10	8	15	21	32
Prefer not to say	1	2	2	4	0	0
Highest educational level						
No qualifications	4	6	0	0	6	9
GCSE/A level	35	52	22	41	15	23
Undergraduate degree	13	19	15	28	16	25
Postgraduate degree	11	16	14	26	27	41
Other	4	6	3	6	1	2
Employment						
In education	11	17	18	34	24	36
Employed	28	42	28	53	38	58
Unemployed	27	41	7	13	4	6
Age	Mean	SD	Mean	SD	Mean	SD
	38.75	13.28	33.78	14.32	31.95	12.69
	Min	Max	Min	Max	Min	Max
	18	78	18	71	19	69

The first group of autistic participants (*n* = 67) were recruited during a randomised controlled trial (RCT) of a psychological intervention for depression and had moderate-to-severe depression (ADEPT; Russell et al., 2020). They were predominantly recruited from clinical settings and clinical databases and are referred to as *clinical cohort* participants. Inclusion criteria for this group included the presence of moderate-severe depression, defined as scores of ⩾10 on the PHQ-9 depression questionnaire; ability to give informed consent; English literacy levels such that they could engage with a written study materials. Exclusion criteria included high risk of suicide; alcohol or substance dependency; untreated epilepsy; history of psychosis; accessed cognitive behavioural therapy within 6 months of participation. Suicide risk was assessed through checking relevant items of the PHQ-9 and Beck Depression Inventory, and any other statements regarding risk to self which were made during baseline assessment for the clinical trial, followed up by risk assessment from a qualified Clinical Psychologist to assess whether risk was such that a high-intensity psychological intervention was required, in which case, the RCT was not suitable for the individual.

The next two groups were community participants recruited through online and in-person adverts via community groups for autistic and non-autistic people: referred to as *autistic community* (*n* = 54), and *non-autistic community* (*n* = 66) participants. Recruitment was from a wide range of sources, for example, social media including Twitter, Facebook and Reddit, and adverts in community centres and public spaces.

Data regarding these participants are also reported in another study (Cooper et al., 2022), which had different aims and hypotheses. These groups of participants were also aged 18 years and older and required cognitive abilities to read the information sheet and provide informed consent, and to complete the survey measures. Autistic participants further had to provide evidence of a professional diagnosis of Autism Spectrum Disorder, as above.

### Ethics

The depression study was granted ethical approval from WALES Research Ethics Committee (REC 3; Integrated Research Application System project ID: 191558) and the community study was granted ethical approval via the University of Bath Psychology Department ethics committee (16-318). All participants read an information sheet and gave fully informed consent to take part. Participants were aware of their right to withdraw from participation and to withdraw their data from the study, and after participating were provided with debrief information including signposting to relevant support services.

### Procedure

In the case of the clinical cohort, potentially eligible participants were invited to participate in the ADEPT RCT through invite letters sent to participants on clinical research databases, and through autism clinical services based in the North East and South West of England. Potentially eligible participants were shown an information sheet and screened for eligibility by the research team, which included checking their autism diagnosis letter. Participants who met inclusion criteria and gave fully informed consent were asked to complete a range of baseline measures of mental health and associated constructs. These were completed either via a tablet or on paper copies which were then hosted on a REDCAP database. See below for the measures used in this study. Participants completed additional measures which were reported by Cooper et al. (2022) and then completed additional procedures linked to the ADEPT study(Russell et al., 2020), which are reported elsewhere.

In the case of the community sample, participants who had seen the study advert approached researchers. Potentially eligible participants were then sent the information sheet and invited for a baseline assessment. At this assessment, after providing fully informed consent and evidence of their autism diagnosis, they completed several questionnaire measures via Qualtrics, including the measures used in this study (see below). For the full procedure including additional questionnaires undertaken by participants, see Cooper et al. (2022).

### Measures

The measures collected for this study are reported here. These were collected either via completion on a desktop computer, tablet, or paper copies of the questionnaires, depending on participant preference.

#### Demographics

All participants completed a questionnaire regarding basic demographic information, including their age, gender, education, employment, relationship status and ethnicity.

#### Insistence on sameness (Adult Repetitive Behaviours Questionnaire; RBQ-2A subscale; Barrett et al., 2015)

A subscale of the RBQ measuring insistence on sameness was administered to all participants. The insistence on sameness subscale measures higher-order repetitive behaviours including routines and special interests. The RBQ-2A is a 20-item self-report questionnaire, and the insistence on sameness subscale has 10 items including ‘Do you insist on doing things in a certain way or re-doing things until they are “just right”?’. Higher scores indicate more repetitive behaviours, and each item is scored on 3- or 4-point Likert-type scales indicating the intensity of each trait. It is a valid measure on which autistic people have higher scores than non-autistic people (Barrett et al., 2015). Internal consistency was acceptable in both autistic (α = 0.78) and non-autistic (α = 0.74) participants in this study.

#### Depression and anxiety (Patient Health Questionnaire Anxiety-Depression Scale; PHQ-ADS; Kroenke et al., 2016)

Anxiety and depression were assessed with the 16-item PHQ-ADS, which is a validated measure combining the widely used GAD-7 and PHQ-9 measures of anxiety and depression into a single questionnaire. This scale has been found to be unidimensional, have good internal reliability, strong convergent validity, and moderate to large construct validity (Kroenke et al., 2016). Higher scores indicate higher rates of depression/anxiety, with mild, moderate and severe cut-offs at 10, 20 and 30 total scores. Internal consistency was good in both autistic (α = 0.86) and non-autistic (α = 0.86) participants in this study.

#### Obsessing subscale of the Obsessive-Compulsive Inventory (OCI-R; Foa et al., 2002)

Three items measuring obsessing were used from the original 18-item OCI-R measure of OCD symptoms. The OCI-R has been used with autistic populations (e.g. Russell et al., 2013), is valid and reliable (Foa et al., 2002), with good discriminant validity (Cadman et al., 2015). The three items of the obsessing subscale are ‘I find it difficult to control my own thoughts’; ‘I am upset by unpleasant thoughts that come into my mind against my will’; ‘I frequently get nasty thoughts and have difficulty in getting rid of them’. Higher scores indicate more OCD symptoms, and each item is scored on a 5-point Likert-type scale (0–4). Internal consistency for the subscale was good in both autistic (α = 0.84) and non-autistic (α = 0.88) participants in this study.

#### Rumination (Rumination–Reflection Questionnaire; RRQ; Trapnell & Campbell, 1999)

The RRQ is a 24-item questionnaire with two subscales: rumination and reflection. We used the 12-item rumination subscale which includes items such as ‘I often find myself re-evaluating something I’ve done’. Items are scored on a 5-point Likert-type scale from 1 (‘strongly disagree’) to 5 (‘strongly agree’), with higher scores indicating higher levels of rumination. The measure has good psychometric properties, including good construct validity and internal consistency (Trapnell & Campbell, 1999). In this study, we found good internal consistency in both autistic (α = 0.88) and non-autistic (α = 0.93) participants.

### Analysis

Three participants were excluded entirely from the clinical cohort due to not having completed measures relevant to this study, leaving the 67 autistic participants in the clinical cohort reported on in this article. Participants recruited for the community study were all included in the analyses. Pairwise deletion was used to exclude incomplete data from the analysis. The assumptions of analysis of variance (ANOVA) and regression were checked (and met) before the analysis was completed.

We conducted preliminary correlations of all study variables, dividing participants on the basis of being autistic or not autistic.

To test hypotheses 1 and 2, we conducted a one-way multivariate analysis of variance (MANOVA) comparing insistence on sameness, rumination and obsessing scores in community autistic participants and community non-autistic participants. This analysis included only community participants to provide a comparable sample to understand differences in rumination and obsessing between autistic and non-autistic individuals.

**Figure 1.**
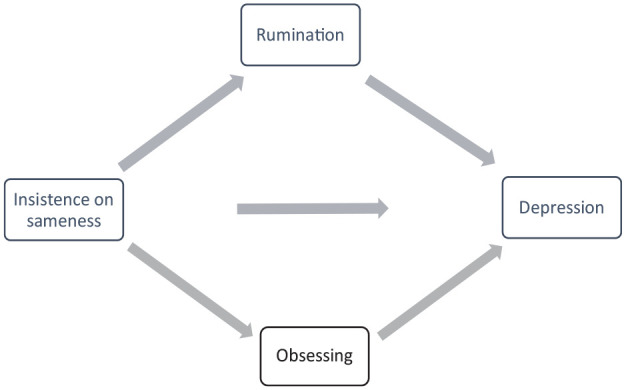
Mediation model to be tested in this study.

To test hypothesis three, we conducted a parallel mediation analysis using the PROCESS macro in SPSS (Hayes, 2017), see Figure 1 for a diagram of the model to be tested. This was conducted with all participants (autistic and non-autistic) to align with the transdiagnostic approach taken in this paper. The predictor variable was insistence on sameness, outcome variable was depression-anxiety, with two mediators of rumination and obsessing. A total of 5000 bootstrap samples were used to calculate percentile bootstrap confidence intervals. The analysis was not pre-registered. All participants were included in this analysis to ensure for a range of depression scores (from the clinical cohort, who were autistic participants with moderate-severe depression) and a range of insistence on sameness scores (from autistic and non-autistic participants), and given the similar pattern of correlations across autistic and non-autistic participants on the key study variables.

### Community involvement

This research was conducted by non-autistic clinicians with experience of working in mental health and university settings with autistic people. There was no involvement of autistic individuals in this research.

## Results

### Hypothesis 1

A one-way MANOVA indicated that autistic community participants had significantly higher insistence on sameness scores (M = 2.28, *SD* = 0.45) than non-autistic community participants (M = 1.42, *SD* = 0.34), with a large effect size *F*(1,119) = 142.64, *p* < .001 (η^2^ = .55).

### Hypothesis 2

Autistic community participants had higher rumination scores (M = 4.11, *SD* = 0.61) than non-autistic community participants (M = 3.21, *SD* = 0.80), with a large effect size, *F*(1,119) = 46.75, *p* < .001 (η^2^ = .28). Moreover, autistic community participants had significantly higher obsessing scores (M = 7.04, *SD* = 3.30) than non-autistic community participants (M = 2.39, *SD* = 2.84), with a large effect size *F*(1,118) = 68.19, *p* < .001 (η^2^ = .37). See Table 2 for descriptive statistics for all included measures.

**Table 2.** Means and standard deviations (SD) for autistic clinical cohort (*n* = 67), autistic community sample (*n* = 54), and non-autistic community sample (n = 66).

Measure	Autistic clinical cohort		Community		Non-autistic community	
	*Mean*	*SD*	*Mean*	*SD*	*Mean*	*SD*
Insistence on Sameness (subscale of RBQ)	2.31	0.42	2.28	0.45	1.42	0.34
Depression-Anxiety (PHQ-ADS)	28.34	7.06	22.30	9.99	7.83	6.10
Obsessing (subscale of OCI-R)	6.23	3.44	7.04	3.23	2.40	2.84
Rumination (subscale of RRQ)	4.11	0.53	4.11	0.61	3.21	0.80

RBQ = Repetitive Behaviour Questionnaire; PHQ-9 = Patient Health Questionnaire; GAD-7 = generalised anxiety disorder; OCI-*R* = Obsessive-Compulsive Inventory – Revised; RRQ = Rumination–Reflection Questionnaire.

### Hypothesis 3

We conducted a preliminary analysis to investigate the correlations between main study variables separately in autistic and then non-autistic participants. All correlations between study variables (insistence on sameness, depression-anxiety, rumination, and obsessive-compulsive inventory) were positive and significant separately in autistic participants (clinical cohort and community sample combined) and non-autistic community participants, with moderate to large effects (see Table 3).

**Table 3.** Bivariate correlations between variables for all autistic participants below diagonal (*n* = 121), and non-autistic participants above the diagonal (*n* = 66).

		1	2	3	4
1	Insistence on Sameness	-	.38**	.44**	.32*
2	Depression – Anxiety	.40**	-	.54**	.62**
3	Rumination	.47**	.42**	-	.67**
4	Obsessing	.38**	.51**	.47**	-

** *p* *<* .001 *p < .01.

To test hypothesis 3, that the relationship between insistence on sameness and depression-anxiety would be mediated by rumination and obsessing, the mediation analysis was conducted by combining the three groups of participants (see Table 4). This mediation model in the full, neurodiverse sample, was found to be significant, as there was a significant indirect effect of insistence on sameness on depression-anxiety via both rumination and obsessing. The results of bias-corrected bootstrapping with 5000 samples indicated that the standardised bootstrapped indirect relationship between insistence on sameness and depression-anxiety via rumination was 0.09 (95% CI = 0.01, 0.18) via obsessing was 0.22, (95% CI = 0.13, 0.31), and in total was 0.31 (95% CI = 0.22, 0.41). The contrast between the two mediators was not significant at −.12 (95% CI = −0.27, 0.03).

**Table 4.** Unstandardised coefficients and 95% confidence interval for mediation analysis of insistence on sameness on depression-anxiety via rumination and obsessing including all autistic (clinical cohort and community) and non-autistic participants (*n* = 182).

Regression effects	B coefficient	95% confidence interval
(a1) Insistence on sameness → Rumination	0.88	0.73–1.03
(a2) Insistence on sameness → Obsessing	3.94	3.18–4.70
(b1) Rumination → Depression-Anxiety	2.14	0.16–4.11
(b2) Obsessing → Depression-Anxiety	1.11	0.72–1.50
(c’) Insistence on sameness → Depression-Anxiety	8.32	5.84–10.81

## Discussion

We set out to investigate if RNT is an important transdiagnostic factor in common mental health problems, that is, anxiety and depression, for autistic and non-autistic adults. We found that autistic adults from a community, non-clinical sample, had higher insistence on sameness and higher RNT scores than non-autistic adults from a community, non-clinical sample.

Our findings highlight the role of RNT in depression-anxiety, consistent with previous research (e.g. Gotham et al., 2014; Williams et al., 2021). We demonstrated that RNT significantly mediated the association between insistence on sameness and anxiety and depression in our participants. Higher rates of insistence on sameness, whether in those meeting criteria for an autism diagnosis, or who experience high insistence on sameness in the absence of an autism diagnosis, increases risk of depression and anxiety, via RNT processes. We measured repetitive negative cognitions in two ways, obsessing and rumination, which were highly correlated. Both had a significant mediating effect but neither a significantly stronger effect than the other.

RNT has been identified as an important transdiagnostic factor for common mental health problems in the non-autistic population. Our findings suggest that the cognitive processes underpinning depression and anxiety for autistic people are like those for non-autistic people, and that these are influenced by restricted and repetitive behaviours across neurotypes. This was observed in our analysis which included non-autistic and autistic people.

These findings indicate that it is not restricted and repetitive behaviour in and of itself that is responsible for elevated anxiety and depression. The content and process of cognitive repetition are key. This is consistent with the important findings of Gotham et al. (2014) reporting that self-perception of autism impairment was significantly associated with depression in the presence of elevated rumination. Negatively oriented thoughts and views about the self, when experienced as repetitive, intrusive and uncontrollable thus increase the risk of significant anxiety and low mood. Rumination is known to predict the number and duration of episodes of major depression (Robinson & Alloy, 2003). However, findings from a large study in autistic adults found that personality factors also mediate the relationship between insistence on sameness and depression in autistic adults (Schwartzman et al., 2022). Further research to unpack the importance of state versus trait factors is needed.

There are multiple psychosocial risk factors for depression and anxiety, to which autistic people may be more susceptible. Contextual factors including access to meaningful and pleasurable occupations and activities, social connectedness and social support and exposure to adverse life events are well-established as important in understanding depression. There is research evidence and first-person accounts about the difficulties encountered by many autistic people in living their everyday lives.

However, our findings add to the growing evidence that the repetitive behaviours domain of autism, specifically insistence on sameness, may increase autistic people’s vulnerability to RNT, an important transdiagnostic factor in depression and anxiety.

### Limitations

Our convenience sample limits the generalisability of our findings to the wider population. Notably, the sample is predominantly White, and the non-autistic community samples were highly educated, with 66% having an undergraduate or postgraduate degree. Further studies with a more representative sample are warranted. Our study design does not allow directionality or causality to be established in respect of the associations between insistence on sameness and anxiety depression. We chose to use the obsessional subscale of an OCD symptom measure as one of our indices of RNT. This limited our mental health measures to anxiety and depression, excluding OCD symptoms from our analysis, although obsessional thinking was included in our measure of RNT. Furthermore, the mediation analysis included all participant groups, so can shed light on the relationship between insistence on sameness traits and depression-anxiety, demonstrating the similarities in processes between autistic and non-autistic participants.

### Clinical implications

Understanding the role of RNT is critical to the development of effective mental health interventions. Interventions which target transdiagnostic factors offer more efficient treatment options. Anxiety and depression are highly co-morbid conditions (Kessler et al., 2007) and a single intervention targeting both has many potential benefits.

Evidence-based interventions for anxiety and depression include cognitive behaviour therapy (CBT) which can place an emphasis on cognitive or behavioural change as key to reducing distress. CBT has been shown to be effective in reducing anxiety for autistic young people if adapted to meet their needs (Wood et al., 2009). Among the recommended adaptations to CBT for autistic people includes emphasising behavioural change over cognitive change (NICE, 2012). While such adaptations may increase the accessibility of CBT for autistic people, not directly intervening with RNT at a cognitive level may reduce the possibility of transdiagnostic interventions and the efficacy of interventions for specific problems such as social anxiety where post-event processing or rumination is considered a key maintenance factor (Clark & Wells, 1995).

Efforts to improve the accessibility of cognitive change techniques of CBT for autistic people are much needed. However, these techniques may be experienced as problematic by many autistic people for whom executive function difference can include reduced cognitive flexibility and reduced fluency (e.g. Demetriou et al., 2018). Indeed, cognitive inflexibility is noted as significantly associated with internalising symptoms in young autistic people in a recent review and meta-analysis on this topic (Lei et al., 2022). If autistic people are vulnerable to RNT and some autistic people are constitutionally less able to shift thinking patterns, interventions that rely on cognitive change may be a poor fit for this group.

Transdiagnostic psychological interventions specifically targeting rumination include Rumination-focused CBT, Cognitive Control Training (CCT) and Metacognitive therapy and Mindfulness-based CBT. Rumination-focused CBT includes functional analysis of rumination, imagery, behavioural experiments and cognitive bias modification (Watkins, 2015). In respect of functional analysis, the approach is based on the principles outlined in Behavioural Activation for depression (Jacobson et al., 2001). Information about antecedent situations and mood associated with rumination is analysed and alternatives scheduled. Behavioural Activation has been adapted for autistic adults experiencing depression and found to be feasible and acceptable (Horwood et al., 2021; Russell et al., 2020). This suggests that at least some elements of rumination focused CBT can be successfully adapted for autistic people. CCT was developed in response to findings that executive function impairments have been reported in individuals with high levels of depressive rumination. As executive functioning (EF) impairments are frequently reported in autistic people across the lifespan, CCT interventions such as attention control training (Papageorgiou & Wells, 2000) may be highly relevant. Social cognition and executive function have been the targets of cognitive remediation interventions for autistic people to date (see Dandil et al., 2020 for a review) with some evidence for effectiveness, suggesting that attentional control training could be feasibly adapted for autism. There is evidence that rumination reduces following treatment for depression in autistic people from a randomised control trial of mindfulness-based stress reduction (MBSR; Spek et al., 2013). Participants were included in the study if they were experiencing rumination, anxiety or depression. MBSR aims to reduce rumination through increased present-moment awareness and therefore decrease depression symptoms. Participants in MBSR had lower depression scores and rumination scores than the control group, and these two phenomena were correlated providing some preliminary evidence that rumination can be improved in autistic people using mindfulness-based approaches.

An alternative approach is to consider the content rather than process of RNT. In the descriptive experience sampling study (Cooper et al., 2022), autistic community participants did not report more negatively oriented thoughts than non-autistic community participants, but they did report more cognitive repetition. Negative, self-oriented thoughts can fuel both low mood and anxiety. Earlier studies indicate that increased awareness of and negative perceptions about autism may have a negative impact on mental health likely through social comparison processes (Cooper et al., 2017; Gotham et al., 2014; Hedley & Young, 2006). Societal-level interventions to support the development of balanced and strength-based accounts of autism for all people may go some way to reducing the development of one important dimension of the content of negative, self-oriented repetitive thoughts, that is, negative thoughts and perceptions related to being autistic.

## Conclusion

There is evidence that a transdiagnostic factor, RNT, is important in understanding the elevated rates of depression and anxiety reported in autism. Psychological interventions targeting the process of RNT may be helpful for autistic people if adapted to meet their needs and offer the possibility of addressing depression and anxiety within a single framework. Interventions at a societal level to counter negative perceptions of autism are also key.
